# Embodied Decision-Making Style: Below and Beyond Cognition

**DOI:** 10.3389/fpsyg.2018.01123

**Published:** 2018-07-04

**Authors:** Brenda L. Connors, Richard Rende

**Affiliations:** ^1^Office of the President, Naval War College, Newport, RI, United States; ^2^Social Behavioral Research Applications, Phoenix, AZ, United States

**Keywords:** embodied cognition, decision-making style, decision-making process, organizational decision making, movement pattern analysis, human movement science, leadership analysis, leadership development

## Abstract

There is growing recognition of the essential role of sensorimotor processes as not just a supporter of the cognitive aspects of decision making, but rather as a foundation for all the coordinated physical and mental activities that go into how we make decisions. We illuminate concepts and methods for examining embodied decision making through the lens of Movement Pattern Analysis (MPA). MPA is as a prime example of a conceptually rooted observational methodology for deciphering embodied decision making and for decoding how people differ as decision makers with respect to cognitive motivational priorities. The historical origins of MPA that predated the formalized recognition of embodied cognition are presented, along with an overview of both the theoretical model and methodology. Advances in research on two psychometric benchmarks of observational research—inter-rater reliability and predictive validity—are highlighted as an empirical platform for the strong promise of MPA as a tool for understanding individual differences in embodied decision-making style. Future directions for research are considered—specifically with respect to the potential for utilizing automated coding, and the need for collaborative neuroscience research efforts—which would support further understanding of how decoding movement patterning captures human motivation at the level of sensory, motoric, cognitive and action integration which drives how people function as decision makers.

## Decision making as embodied cognition

In non-academic circles, when we talk about decision making, our language gravitates to discussions of thought processes. We reference how we weigh options, if we waffle or pull the trigger, if decisions are no-brainers or extremely hard. The emphasis is on the “decision” as something we do with our mind, the range of decisions we are faced with, and the different types of thinking that help us make decisions.

Much of the classic literature on decision making has taken a similar perspective. In terms of theory, decision making has been dissected into a number of cognitive processes. Research has brought much insight into how these processes are utilized to make decisions, typically under different types of demands, ranges of complexity, and varying rewards and consequences (e.g., Connors et al., 2013, 2018a).

While we have learned much about the cognitive side of decision making, there are still major gaps to be filled in terms of understanding how people enact decisions in real world settings. One key consideration is that decision making, like other forms of cognition, actually involves much more than cogitation. Here research on decision making is aligned with the thrust of work linking the body to thought, as articulated across social science domains and collectively known as embodied cognition. That said, while there are nearly two decades of scholarly papers devoted, formally, to embodied cognition, most do not focus explicitly on decision making.

That is starting to change, and for good reason. People are biologically wired not just to think, but also to determine when, why, and how to act in relation to changing internal and external physical, emotional and social surroundings. There is an inherent “sensorimotor coupling” between person and environment that defines the essence of embodied cognition^1^, as expressed as changes in observable indicators such as facial expressions, postures, and gestures (Pietrazak et al., 2018). With respect to decision making, we are seeing a current of recognition of the essential role of sensorimotor processes as not just a supporter of cognition, but rather as a foundation for all the coordinated physical and mental activities that go into how we make decisions (Connors et al., 2018a). For example:

“The central statement of embodied choice is the existence of bidirectional influences between action and decisions. This implies that … the action dynamics and its constraints (e.g., current trajectory and kinematics) influence the decision making process” (Lepora and Pezzulo, 2015, p. 1).

In this paper, we illuminate concepts and methods for examining embodied decision making through the lens of decoding signature movement patterns. We focus on Movement Pattern Analysis (MPA)—including its historical origins that predated the formalized recognition of embodied cognition—which serves as a prime example of a conceptually rooted methodology for deciphering embodied decision making.

## Decoding movement as insight into embodied decision making

Our point of departure is to focus intensively on direct observation of the body's patterning of movement to provide unique insight into decision making. We suggest that human movement underlies the inherent connection between thinking and behavior and resides “below and beyond” cognition. Within the sphere of embodied cognition, it has been well appreciated that movement supports cognitive functions (and hence is “below” cognition) by being a mechanism responsible for orienting the body to take in sensory information and facilitate perception, and to translate thought into action. That said, we wish to promote a deeper principle—that patterning of movement captures *human motivation* at the level of sensory, motoric, cognitive and action organization that drives how people go about making decisions (and hence is “beyond” cognition).

This principle has long roots and dates to the innovative insights of pioneers in movement analysis as it relates to human cognition and behavior, which we have reviewed in depth (Connors et al., 2018a) and hence summarize here. The Hungarian polymath Rudolf Laban (1878-1958) was a movement theorist and father of movement analysis and notation. A visual artist as well as dancer and choreographer, his acute observations of dance led him to decipher how movement conveys inner attitudes and the expression of psychophysical and emotional cues (Laban, 1950). Importantly, this led him to devise systematic ways to decode movement via two systems of analysis (which we would now call observational coding systems). Labanotation or *Kinetography Laban* is a notation system for recording and analyzing human movement. Analogous to music notation, it records complex actions of the body and dynamic nuance via symbols. Notating the movement and expression provided a way for dance choreography to be reproduced from a written score (Laban, 1928).

Notably, Laban also created Laban Movement Analysis (LMA), which is a multidisciplinary method and language for describing, visualizing, interpreting and documenting all varieties of human movement. LMA is particularly relevant for the purposes of this paper, as it eventually became an innovative platform for observing decision making via the coding of movement in naturalistic settings (Moore, 2005; Lamb, 2012; Connors et al., 2018a). In brief, in 1941 Laban was in England (as a refugee from Nazi Germany), where he was recruited to collaborate with F. C. Lawrence (an engineer and time motion expert) to increase productivity of women on factory assembly lines (who were working there as men were serving in the war). Laban pioneered an “Industrial Rhythm” approach that apprehended and honored the unique rhythmic patterns of workers, which yielded many positive results in the factory. Importantly, Laban and Lawrence (1947) also expanded the method to study clerical and managerial workers as they performed their duties. Here they detected distinctive movement patterns that corresponded to different types of white collar jobs and tasks that would not typically be considered to be physical in nature, providing, in the naturalistic setting, an insight and perspective consistent with embodied decision making.

It is particularly important to recognize that the detection of movement patterns came from observations of individuals moving freely in their naturalistic (work) environment. This provided an authenticity—what we would refer to today as ecological validity—that drove him to devise meaningful systems for recording movement and notating a number of complex patterns that corresponded to psychological processes. This point also reinforces the role of the highly trained and sophisticated human observer, who is capable of parsing what is apprehended as observable into systematic recording systems that capture the deeper significance of movement patterning. We will return to these two points later in this paper.

## Movement pattern analysis (MPA): embodied decision making

One of Laban's protégés, Warren Lamb, was brought in and was trained to examine in more depth the relations between movement patterns and job responsibilities. Lamb continued this work and observed how distinctive patterns of movement corresponded to higher-order functions, including decision-making processes. This led Lamb to formalize his intensive observations by developing a conceptual model and corresponding coding system for recording signature movements that align with stages of decision making, collectively referred to as MPA. Lamb's approach included a number of notable features (e.g., Moore, 2005; Lamb, 2012; Connors et al., 2018a), each of which illuminate his prescient abilities as an observer of human movement as they are key elements of current thinking in decision-making science.

First, Lamb's observations led him to conceptualize decision making as involving three *distinct stages*. These are as follows:

- *Attending*: Engaging in the preliminary consideration (e.g., of information deeply and broadly) that goes into making a decision knowledgeably.
- *Intending*: Coming to a resolute ascertainment of the best course of action driving the decision to be made.
- *Committing*: Mobilizing to implement both tactically and in longer term staging what has been intended; initiating and pacing action adroitly to attain goals and avoid pitfalls.

Lamb also proposed that, within each stage, individuals have a need to coordinate two complementary goals, or motivations, that drive the overall decision-making process. These are known as the Overall Factors in the MPA model, and are as follows:

- *Assertion*: Applying effort or elbow grease by focusing, pushing and pacing tactically to make the decision happen.
- *Perspective*: Positioning the body to create an environment in which to reflect on the broader strategic context and how parts fit together.

The approach observes and records signature movement patterns that corresponded to each stage in the MPA model, and which reflect either Assertion or Perspective. Lamb focused on a specific movement phenomenon that was the platform for the body to engage in a range of decision-making processes—the Posture-Gesture Merger, or *PGM* (Lamb, 2012). A PGM reflects the coherent integration of both a gesture (an action that is isolated to one or two body parts, such as head nodding or a foot and thigh crossing) along with a postural action involving all body parts (such as the whole body moving in a head-to-toe jump to get someone's attention) (Moore, 2005; Lamb, 2012; Connors et al., 2018a).

The MPA model identifies PGMs that are organized along the two interrelated Overall Factors in relation to the three planes of motion (horizontal, vertical, and sagittal) (Connors et al., 2018a), They are hence decoded to reveal how a person effortfully applies energy to investigate, push or pace (Assertion) vs. how in the kinesphere around the body a person shapes to explore, prioritize, and anticipate (Perspective). PGMs have been shown to be generated spontaneously by individuals in conjunction with verbalizations that reflect authenticity (Winter et al., 1989; Winter, 1992; Lamb, 2012), suggesting that they are reflective of meaningful thought and action.

Lamb discovered that there are a variety of PGMs that correspond to the stages in the MPA decision-making model, and which align with either Assertion or Perspective. He continually refined this discovery diving deeper into its essence of the two Overall Factors and the three stages of decision making and uncovered six distinct Action Motivations which reflect the two types of Overall Factors at each stage, one motivation reflecting Assertion, and one motivation reflecting Perspective. Each of the Action Motivations can be discerned by the highly trained observer by detecting signature PGMs (see Connors et al., 2013, for more details):

- During Attending, individuals engage in *Investigating* (applying effort, or hence Assertion, to scan/probe/analyze relevant information within a prescribed area) and *Exploring* (positioning oneself, to gain hence Perspective, and to be open to a wide range of ideas along with potentially relevant resources). In terms of PGMs, an example of Investigating would be an individual zeroing in on a target (e.g., a map) with head and torso, merged with a pointing gesture; an example of Exploring would be spreading the chest and upper torso broadly while having that movement flow into an arm gesture.
- During Intending, individuals can engage in *Determining* (devoting effort, or hence Assertion, to build the resolve necessary to formulate a position and take a stand) and *Evaluating* (positioning oneself, to gain Perspective by sizing up options and setting priorities). In terms of PGMs, an example of Determining would be pressing the body (including hands and feet) down in a chair followed by and merging into gesturing with the chin; an example of Evaluating would be to rise up on one side of the body, crossing the legs and flowing into the “thinker” pose with arms crossed and one hand supporting the head.
- During Committing, individuals can engage in *Timing* (applying effort, or hence Assertion, to implement the decision at the right moment) and *Anticipating* (positioning oneself to gain Perspective in a strategic way to guide and monitor the implementation of the decision staging). In terms of PGMs, an example of Timing would be a very quick forearm and hand gesture followed by the whole body shifting rapidly; and example of Anticipating would be advancing the chest forward and having that movement flow into an head gesture in a meandering way.

The MPA model thus provides a detailed framework for understanding embodied decision making by articulating the distinct stages and observable movement patterns that reflect how an individual interacts with the motion factor and engages in complementary management decision-making processes during each stage.

## Movement pattern analysis (MPA): embodied decision makers

Importantly, the MPA model devised by Lamb also offered futuristic insight into how observation of movement patterning could help us understand *decision makers*, not just decision making. Lamb discerned that while the MPA model identifies universal mechanisms by which we engage in embodied decision making, individuals differ in the extent to which they prioritize and sequence the specific processes. In terms of the stages, people vary in how much they “favor” Attending, Intending and Committing (e.g., some may be “high committers,” whereas others may be “low committers;” some may begin with Committing while others may nearly skip over implementing all together). A fundamental window into how people differ as decision makers comes from how they achieve a balance between the complementary Overall Factors of Assertion and Perspective. Lamb postulated that how each individual achieves their own personal mix of Assertion and Perspective—within and across stages—reveals their cognitive motivational predispositions as a decision maker.

As such, MPA serves as a unique method for capturing *decision-making style* as it is used to provide insight into how each individual goes about making decisions. This is style in the sense that it is not focused on what decisions people make, but rather the way they go about navigating them in terms of balancing Assertion and Perspective across the distinct stages of decision making. MPA has been used in this way for over 50 years by organizations to understand decision makers as leaders, guide selection and placement of top personnel, and inform the building of management teams (Connors et al., 2018a). It has provided the type of ecological validity that Laban and Lawrence (1947) achieved in their pioneering work in the factory and the office.

For the purposes of this paper, we focus on more recent efforts to determine, through the lens of research, the utility of MPA as a method for decoding decision-making style. The vast majority of methods that have been used, in the field, to measure decision-making style are rooted in self-assessment, in the form of questionnaires and inventories (Connors et al., 2013, 2016). While self-perception is an important aspect of decision-making style, there are limits to it; for example, there may be disconnects between how people perceive themselves as decision makers, and how they function as decision makers. Furthermore, there is much interest in moving beyond self-report by focusing on observational approaches that can dig deeper into decision-making processes and pinpoint with more precision how people differ as decision makers (Connors et al., 2016).

Such a consideration is especially important in terms of the types of scenarios that decision makers face. Consider, for example, leaders who navigate complex and ambiguous decision-making situations including crises, and orchestrate time-sensitive and high-stakes negotiations. A key idea here is that there is no one optimal decision-making style and no one way to make a decision; leaders who are high in Assertion or high in Perspective can be equally effective in the same position. That said, having methods that can help leaders understand themselves as decision makers would undoubtedly help them optimize their performance along with improving how they function with their colleagues (e.g., by including decision makers on their team who offer a complementary decision-making style in order to provide better overall balance on issues and crises).

While the long history of MPA in application to organizations certainly serves as proof of principle of its utility, even more confidence in the method would be gained via empirical study. To that end, we now turn to summarizing recent advances in evaluating core psychometric properties of MPA, particularly benchmark indicators including reliability and validity that are evaluated for any system of measurement rooted in observation (e.g., Dishion and Synder, 2004; Rende et al., 2009; Slomkowski et al., 2009; Bakeman and Quera, 2011; Girard and Cohn, 2016).

## Is MPA reliable across expert observers?

Highlighting the many intellectual and methodological contributions of visionaries such as Laban and Lamb illuminates the complexity of mastering MPA coding.

Applying MPA requires decoding integrated patterns of motion infused with degrees of pressure, acceleration, focus, and energy that are manifested in physical space and distributed across temporal parameters in a continuous and spontaneous display throughout the whole body. Doing this right is a hard-earned skill that goes beyond detecting isolated and truncated behaviors. Recognizing overall patterns of movement demands acuity at coupling quantitative and qualitative measures that are sensitive enough to offer discrimination across a wide range of decision-making styles.

To this end, MPA analysts go through rigorous training to develop the expertise necessary to skillfully decode signature movement patterns, and are certified as such. Training includes mastering an advanced level of observational expertise as well as learning how to administer the standard protocol to conduct a full MPA profile, which is executed in a semi-structured interview. The goal is to engage the interviewee in a discussion of his or her career and biographical history, which provides a naturalistic platform to observe their embodied decision-making motivations and priorities. The interview typically requires 90–120 min to allow the analyst to gather a sufficient sampling of PGMs to provide confidence in establishing an individual's signature patterning with respect to the stages of decision making, Overall Factors, and Action Motivations. Coding is done during the observation in real time, and can also be undertaken via review of a videotaped recording of the interview.^2^ Importantly, the certified MPA analyst is also trained to determine, much like a clinician, if and when they have observed a sufficient sampling of PGMs to feel confident that they have acquired a representative baseline for determining an individual's propensities. A final aspect of the assessment process is interpreting and writing in words the findings with a refined degree of balancing the many variations in movement that are quantified in the coding into a coherent picture of propensity and pattern.

The total number of PGMs provides a “denominator” for each individual, such that their movement patterning can be represented as percentages out of a grand total of 100 per cent reflecting the PGMs expressed as Assertion or Perspective within and across the stages of the MPA model (as reflected in the Action Motivations). This self-referenced balancing in percentage terms provides an elegant quantitative representation of their decision-making priorities and motivations.

### Methods for gauging inter-rater reliability of MPA

Given the richness and specificity of MPA, a key issue, and a requirement for any observational system, is gauging the inter-rater reliability of MPA, which informs us of the extent to which different trained experts converge on their assessments of an individual's decision-making profile. In general, gauging reliability can be a formidable task when the methodology demands high precision in separating signal from noise in the stream of real-time behavior, as is the case with MPA. Moreover, MPA is unlike many other nonverbal behavioral coding systems that specialize primarily within one subsystem and code all relevant movement behaviors in that area (e.g., gesture or facial expression). Indeed, MPA is distinctive in that two areas of movement repertoire or subsystems—namely the posture and gesture of movement—are involved that ultimately result in a merged quality throughout the entire body. These synchronized movements merge and flood into a continuous stream of energetic activity from head-to-toe.

The implication is that calculating inter-rater reliability of MPA is not just a straightforward exercise of determining if different raters agree when coding the behaviors of interest, namely, the categories of PGMs. One standard approach used in observational research would be to measure the degree to which different coders agreed on every rating that was made during the observation with a degree of time anchoring (e.g., some window of time applied to any event detected by a rater, during which time another rater would be in agreement if they also recorded the event). Here percent agreement across raters would be calculated, and inter-rater reliability would be calculated using indicators that correct for chance agreement, such as Cohen's Kappa. An alternative would be to aggregate totals of PGMs within categories and then assess inter-rater reliability on these quantitative measures using the Intraclass Correlation Coefficient (ICC). An issue, however, is that neither of these conventional strategies would be aligned with the nub of MPA coding. MPA is oriented toward the patterning of target behaviors *within each subject* (rather than total counts of behaviors), which is thought to best reveal decision-making propensities.

We have framed such considerations as an empirical issue, as we compare the level of reliability of coding individual patterning of signature movements, to the level of reliability for raw count tabulations. For each individual, we would have a *total* number of PGMs coded as reflecting either Assertion or Perspective (raw counts), as well as a *proportional representation* for each subject that captures the balance between these Overall Factors relative to each individual's total number of PGMs (individual patterning). A hypothetical serves to make the issue more transparent. Consider two individuals: subject one who had 100 PGMs coded as Assertion, and subject two who had 120 PGMs coded as Assertion. Based on these numbers, it is not possible to conclude that subject two is more inclined to Assertion as compared to subject one. What is missing is the denominator, or the total count of all PGMs (those coded as either Assertion or Perspective). For example, if subject one had a tally total of 140 PGMs, then about 70% of this individual's PGMs (100/120) would indicate Assertion. If subject two had a total tally of 210 PGMs coded, then nearly 60% of this individual's PGMs would indicate Assertion (120/210), which is lower than the 70% for subject one. We would thus conclude, in terms of individual patterning, that subject one was more inclined to Assertion than subject two, even though subject two had a higher absolute number of Assertion PGMs.

Using this approach, we computed inter-rater reliability using two different indicators of how each individual expressed Assertion and Perspective in their movement patterning. One indicator focused on the *relative percent* of Perspective and Assertion as referenced by each individual subject's own baseline and as such labeled as a P/A Balance Score (Connors et al., 2014). The P/A Balance Score is calculated as [% Perspective – % Assertion] and conveys the conceptual point that MPA determines the relative importance that a person allocates to each of the Overall Factors, which is displayed in how they move unconsciously to each motivation factor as a decision maker. A value greater than zero indicates more emphasis on Perspective; a value less than zero represents more emphasis on Assertion; and zero signals equal emphasis on both Assertion and Perspective. We also computed a similar difference score for the *raw counts* of PGMs coded as Assertion and Perspective—calculated for each subject as [# PGMs Perspective – # PGMs Assertion]—which we termed a P/A Difference score, to represent each individual's balance between the Overall Factors through the lens of total PGM counts. In Connors et al. (2014), we reported a range of values for both the P/A Difference score and the P/A Balance score in a sample of leaders, reflecting, as expected, individual differences across the subjects in terms of their cognitive motivational style as captured by MPA and the framework for decision making.

As discussed in Connors et al. (2014), inter-rater reliability was substantially higher for the patterning of PGMs within subject as detected by the P/A Balance Score (ICC = 0.89, CI = 0.77–0.95) as compared to the raw counts of PGMs (ICC = 0.41, CI = 0.02–0.69). A comparison of the 95% confidence intervals for these ICC coefficients reveals a lack of overlap, providing evidence that the difference is statistically significant.

### Pattern decoders

We reason that this comparison reveals a major point about observational methodology in general, and in particular the distinctive capture made in movement analysis. The strength of the MPA model is that it zeroes in on movement patterns indicative of embodied cognition—authentic, real-time actions that support and represent higher-order thought. The utility of the MPA model and coding system, and the skill of the expert MPA analyst, comes from training and experience in being highly attuned moment-to-moment to each individual's personal baseline and its relative balancing of the complementary decision-making processes. One could argue that in making these observations astutely the total raw counts are not as important as deciphering the stable patterning demonstrated by the individual within the interview setting.

The analogy is a clinician who knows how to get exactly the right amount of data when interviewing a patient to make a diagnosis, as in the case of administering a semi-structured interview for a psychiatric disorder such as depression. While the interview yields quantitative information (e.g., length of time feeling depressed, ratings of symptom severity, symptom counts), there is a profound qualitative element in the sense of the clinician intuiting how best to arrive at the formulation required for that purpose—and in fact knowing just when it is that sufficient information has been acquired to finalize a diagnosis. The same holds for the MPA expert analyst, especially in terms of deciphering the inimitable way each individual displays signature phrases that replicate throughout a full observation period.

The broader point is that the micro coding of the PGMs represents a quantitative method that serves to guide the expert MPA analyst in deducing qualitative patterns that exhibit a macro understanding of an individual as a decision-maker. In short, the PGM Action Motivations also point to levels of Assertion and Perspective as well as sequencing—just how the decision maker is staging their cycle of decision making—whether for example they begin at Committing and then Intend and finally Attend or more locally Attend, Intend and Commit (see Connors et al., 2018a). This blending of the micro and macro and of the quantitative and qualitative is emblematic of the deep method of MPA analysis—the sensitivity to the patterning of movements within each individual is what expert analysts are trained to see—and thus the MPA analyst is adept at revealing an individual's management style.

## Does MPA show evidence of predictive validity?

Establishing high inter-rater reliability of a coding system ensures that raters use it in a similar, consistent manner. This does not tell us if the coding system is performing as it is designed in order to predict future behavior. In the case of MPA, which is predicated on decoding embodied decision-making style, we would want to know if the reliable MPA profile is capable of offering prognostic value for how people will make future decisions at other times and in other settings. In order words, we would need to establish the predictive validity of MPA, which is the essence of establishing an empirical basis for using it to decode cognitive motivational decision-making style.

This brings us to the interdisciplinary framework necessary to evaluate the extent to which MPA offers predictive validity. We have aimed to build a bridge with decision-making science in order to identify ways of measuring what MPA was designed to predict, namely how people will stylistically go about making future decisions, and the corollary of being able to discriminate how a group of people will vary in this process. Here we outline steps that we have taken in our experimental work over the last 6 years that integrate constructs and measures from other disciplines to pursue this interdisciplinary aim (see Connors et al., 2013).

### Measuring individual differences in decision-making processes

A fundamental consideration was to work out the best way to measure individual differences in decision-making processes that could be recorded in real time and serve as the dependent variable to be predicted by MPA. A review of the prior literature at the time (see Connors et al., 2013) stimulated key considerations that guided our work. A primary recognition is that many paradigms used to directly study people as they engaged in decision making were highly constrained and as such would tend to diminish, rather than expose, differences in decision-making style (Connors et al., 2018a). We summed this up as follows:

“ … many of the experimental methods used to study decision-making behavior can overwhelm or diminish the impact of individual differences—meaning that other designs need to be entertained. For example, very detailed instructions, strong manipulations within a paradigm, and highly restrictive forced choices (especially two-choice options) can dilute the role of personal characteristics in the experimental setting. It is critical that research methods be employed that can better simulate the real-world context of decision-making …” (Connors et al., 2013, p. 3).

We turned to a paradigm that has been used across different disciplines: the design and application of hypothetical decision-making scenarios that can be administered in a laboratory setting. A key consideration was that the interest was not in what decisions were made *per se* but in how individuals went about making them. Hypothetical scenarios could be utilized for this purpose, particularly if we attended to a fundamental design principle—subjects need to be granted some form of control over the information they could utilize to inform their decision making, and time constraints needed to be removed:

“To this end, we permitted subjects the freedom to control their own information search via the option of making requests for more information … as it is assumed that decision style would be influential in shaping this aspect of the process … decision style should be reflected in the strategies and motivations that guide information search (as some individuals would lean toward acquiring more versus less information before coming to a decision) as well as response time (as those who seek out more information would also spend more time before coming to a decision)” (Connors et al., 2013, p. 4).

The parameters referenced in the above quote—information search and response time—may be considered as indicators of the predecisional stage of decision making that may be especially resonant of individual differences in decision-making style when recorded while subjects engaged in hypothetical scenarios which offered them control over information and no time constraints. Importantly, we proposed such predecisional stages as articulated in the MPA model and captured by the coding system—again, the core is the manner in which individuals approach decision-making situations, particularly in terms of the priorities and cognitive motivations that define their stylistic propensities.

To generate outcome measures to gauge predictive validity, we created hypothetical decision-making tasks representing four domains (Financial, Health, Voting, and Strategy) drawn from prior work in political science (e.g., decision tree modeling) as well as behavioral research (see Connors et al., 2013). To facilitate expression of individual differences, subjects were provided options to request, one at a time, an additional piece of information to consider before they registered their decision. Subjects could either move on to make a final decision, or request another piece of information, in an iterative manner, at each step. In this way, the number of information requests (information search) could be tallied for each subject, and the amount of chronological time (decision time) spent before a decision was reached could be recorded.

### Analytic approaches and findings

By crossing these two different methodologies across time—MPA as a baseline measure of the cognitive motivational decision style, and laboratory recordings of information search and decision time via the hypothetical scenarios—we had a platform to examine the predictive validity of MPA.

Our first benchmark paper revealed that the P/A Balance score was a robust predictor of individual differences in leaders' decision-making processes, as elicited in the decision-making scenarios. Specifically, a propensity for more Perspective was highly correlated with requesting more pieces of information and devoting more chronological time before coming to a decision (Connors et al., 2013). The correlations were in the “high” effect size range, suggesting robust prediction given the expectations in social science research.

We next examined the relative predictive values of the P/A Balance Score as compared to that offered by the P/A Difference score. In other words, we wanted to determine if the superior reliability of the patterning captured by the P/A Balance Score offered stronger and independent prediction of future decision-making processes as compared to raw count tallies of PGMs. This was indeed the case, as confirmed in stepwise regression models (Connors et al., 2014). The implication is that it is most informative to understand each individual's unique patterning of signature movements—how they balance their predilections for Assertions vs. Perspective relative to their own baseline of total PGMs—rather than absolute counts of PGMs as distributed when gaining prognostic insight from the MPA profile. The “balance” within an individual is the essence of decoding cognitive motivation as it applies to decision-making style.

By utilizing the strategy of creating balance scores using MPA data, we also showed that the individual differences in MPA that were most strongly predictive of the outcome measures could be localized within specific stages of the MPA model (Connors et al., 2015, 2018b). Initial work revealed that the way leaders differed in the way they balanced Assertion and Perspective during the 2nd Intending stage of the MPA model was especially predictive of individual differences in the indicators of predecisional processes measured during navigation of the decision-making scenarios. In particular, leaders who lean toward more Perspective via Evaluating (positioning oneself to size up and crystallize the options and set priorities) requested more pieces of information and devoted more chronological time to the scenarios before coming to a decision, as compared to leaders who were more predisposed toward Assertion via Determining (devoting effort to build the resolve necessary to formulate a position and take a stand). A follow-up investigation utilized a larger sample size to not only replicate these findings, but also utilized factor analysis to show that individual differences in the balance between Assertion and Perspective most predictive of future decision-making processes were localized to the Intending and Committing stages in the MPA model (vs. the Attending stage), providing specificity in terms of how leaders differ in the cognitive motivations that drive the decision-making cycle.

The empirical work done to date complements the decades of application of MPA that has yielded ecological validity within organizations (Connors et al., 2018a). MPA is proving to deliver what is required of measures of decision-making style—significant prediction of how people, including leaders, differ in how they will navigate ambiguous and complex decision-making situations. Leaders across many types of organizations (e.g., corporate, public service, military, academia) face increasing challenges in the types of decisions to be made, the range of information to consider, and the relative stakes attached to their decisions. It has been posited that observational measures of decision-making style would offer prognostic power that transcends the typically moderate prediction provided by self-assessment instruments (Connors et al., 2016), and the findings to date on MPA are certainly in line with that assertion.

## Future directions

It is our hope that new phases of research will be launched that will broaden the interdisciplinary application and study of MPA. Movement resides at the intersection of thought and behavior, and we suggest we can discover deeper truths about what is meant by the term “embodied” as methods like MPA become incorporated into a range of disciplines. Here we focus on two potential future directions that we perceive to be of immediate interest.

### Automated coding

One question that can be raised is the extent to which observational methods such as MPA which focus on detection of complex movement patterns should be facilitated by automated coding. This is a complex issue which is of high relevance because there is a substantial literature devoted to the evolution of a number of automated methods designed to recognize and code nonverbal behavior, including human movement, particularly behavior indicative of psychological processes. Here we break out the issue in a number of ways to stimulate further thinking.

First, there are assumptions that automated coding is inherently superior to coding done by human observers, no matter how highly trained and skilled they may be. For example, there have been suggestions that nonverbal behavioral coding systems executed manually by human observers—including those designed to recognize and code a range of gestures—can suffer from low reliability and a substantial or complete lack of objectivity (Lauberg and Sloetjies, 2016; Mahmoud and Robinson, 2016). While we do not underestimate the challenges involved in coding movement, we do not agree that human observers are fundamentally non-objective and machines are inherently unbiased. Every coding system is, by design, selective, in that specific behaviors are prioritized and coded, and others are ignored, whether the coding is done manually or by machine. Such selectivity needs to be rooted in theory and baseline study, and articulated to a sufficient degree that humans and/or machines can detect the full range of movements as specified in the coding system. Thus reliability becomes the metric of interest, and both humans and machines need to be evaluated empirically to determine the level of “objectivity” of a coding system. With respect to MPA, we defer to our prior section on inter-rater reliability as proof of principle that very high reliability of human coding can be achieved with sufficient standards of training of expert analysts. In other words, certified MPA analysts can achieve the type of objectivity that is desired in automated coding.

A complementary perspective on machine coding of nonverbal behavior, including gesture, should also be considered, as it flips the “bias” in terms of human vs. machine coding. It has been suggested that while much progress has been made in machine recognition of nonverbal indicators of cognitive states (Mahmoud et al., 2016), automated coding systems are often deficient in terms of predictive validity for a range of psychological functions (Lauberg and Sloetjies, 2016). This is a critically important point, in that it reinforces the complexity of detecting the meaning in human movement as it unfolds in real time, and the challenge of designing a machine to achieve the insight of highly trained human observers with decades of experience. We posit that the human element—the level of knowledge and insight offered by expert MPA analysts—will remain essential, especially as it would be what is transferred to a machine. We refer back to the essence of the MPA method, which requires the coding system to detect the signature patterns of an individual. What might prove to be prohibitively difficult for an automated system is generating the baseline—the qualitative understanding of the one-of-a-kind patterning of an individual's movement as it emerges across time and context. The experienced MPA analyst will be able to bring a clinician's rigorous observational experience and deep insight to recognize if a sufficient representative patterning of PGMs has been achieved to permit a valid MPA analysis of a given individual. There are times, for example, when an individual will not produce enough PGMs to permit the creation of an MPA profile (Lamb, 2012), and which may prove impossible to determine by an automated coding system. In addition, when applying MPA, the expert MPA analyst offers cogent interpretation and coaching in tune with the generated profile (Connors et al., 2018a), and that is an essential human skill and deliverable of the MPA process which cannot be automated. As we have shown, this level of insight translates into high predictive validity of how leaders differ in their navigation of decision-making scenarios, and any translation of a complex coding system such as MPA would need to be shown to achieve the same result.

Taken together, we suggest that it is not essential for coding systems such as MPA to be executed by machines in order to achieve the objectivity, reliability, and predictive validity we would demand. That said, the idea of moving toward automated coding of MPA should not be dismissed and in fact should be entertained in future research. The reality is that meaningful observational systems of complex behaviors such as movement patterns requires much time and effort on the part of highly trained human coders (Velloso et al., 2013; Schreer et al., 2014; Mahmoud and Robinson, 2016). It could be advantageous to facilitate the application of MPA to explore methods for integrating automated methods that might be implemented without eliminating the essential expert perspective; such efforts could, for example, permit either more rapid coding of an MPA interview along with facilitating broader application to larger numbers of individuals. While we posit that the complexity of detecting PGMs—highly integrated movement patterns that require perception of the whole body in motion—will prove to be a substantial challenge for developers of automated coding systems, there are studies which have shown initial levels of success at representing the body movements of a samba dancer (Chavoshi et al., 2015), along with application of LMA to detect hand movements (Lourens et al., 2010) and segmentation of a repertoire of motions (Bouchard and Badler, 2007).

One area that could be impactful would be to utilize expert human coding to scan and review videotape of a subject and isolate particularly meaningful segments that could, in principle, eventually be coded by machine. An experienced MPA analyst is skilled in detecting if an individual is revealing notable replicable patterning—particularly with respect to “peak performance,” a patterning akin to an athlete observed with all cylinders firing—which affirms the patterning and finalization of an MPA profile. Future research could work through methodological designs to determine if automated coding of such peak performances would yield the same level of reliability and validity achieved by MPA analysts. If this proves to be the case, this could facilitate more rapid coding of MPA, which could support time-sensitive demands as well as broader application of the method by reducing coding time.

### Neuroscience and different elements of decision-making style

A second core area we envision to be of high importance would be future collaborative efforts that incorporate neuroscience perspectives to expand our understanding of decision making as a fundamentally embodied human behavior. Many advances have been made in deciphering complex neural networks that underlie the cognitive (or executive) functions that go into the decision making process (Rosenbloom et al., 2012). Insight is also accruing on how people differ in the way they make decisions using a range of neuroscience methods (see Connors et al., 2016). For example, Talukdar et al. (2018) focused on seven intrinsic connectivity networks (ICNs) that showed promise as a framework for understanding how individuals differ across multiple components (executive, perceptual, social) of decision making.

There has also been attention given to neural models of embodied cognition. However, these have tended to focus more on neural activation of motor systems in relation to cognition—such as via study of the mirror neuron system (Keysers et al., 2018) and how sensorimotor areas are involved in the processing of action words (Gallese and Cuccio, 2018; Horoufchin et al., 2018)—rather than take on the question of how movement integrates with cognition at the neural level. That said, there have been advances in neuroscience, at the level of findings and conceptual thinking, which certainly converge with the notion of moving toward a neuroscience of embodied decision making.

Perhaps most prominently, current conceptions of the functional capacity of the cerebellum—the “motor control” center of the brain—have uncovered the multiple functions mediated by this structure, which include affective/emotional processes along with perceptual/cognitive operations, echoing the Latin origin of the word cerebellum (“little brain”). Of particular note is that authoritative consensus statements have been made by leading neuroscientists recognizing that the cerebellum plays a much larger role than has been assumed in both perception (Baumann et al., 2015) and cognition (Koziol et al., 2014). As researchers focus more extensively on the cerebellum—both in terms of the range and complexities of functions it subsumes, and the structural and functional connections with subcortical and cortical networks—much deeper insight into how people are wired to be embodied decision makers may be achieved.

A primary impetus for the expanded conception of the functions subsumed by the cerebellum was clinical observation of cognitive deficits in patients suffering from a range of neurological disorders that, in principal, are strictly movement disorders. It became clear, based on a critical mass of clinical studies, that pathology of movement areas in the brain can compromise cognitive capacities, providing proof of principle that movement and cognition are not just functionally connected but also integrated at the neural level. A similar viewpoint is emerging in research on Parkinson's disease, particularly in terms of decision making. Perugini et al. (2018) noted that decision making is often impaired in people with Parkinson's disease, including disruption of the ability to gather a sufficient amount of information necessary to make a decision. They suggest that it will be fruitful to apply a decision-making framework to gain a better understanding of the intersection of motor and cognitive difficulties—including emphasis on the neural circuits that subsume perceptual decision-making and integration with memory—that manifest in the trajectory of Parkinson's disease.

This idea of integration of multiple sensory systems in the brain may hold particular promise for catalyzing our capacity to unravel the profundity offered by embracing the embodied nature of cognition, including decision making. Pasqualotto et al. (2015) emphasize that the recognition of the fundamental nature of multisensory integration involves the interconnections between the “brain, body, and world” which serves as an elegant way of encapsulating why decision making should be conceptualized as an embodied form of behavior. A glimpse into the future is offered by Ryu and Torres (2018), who presented both a theoretical and statistical framework to guide explorations of the integration of biophysical activity with cognitive activity when engaging in decision making. Their novel work revealed, for example, how increases in cognitive load during decision making led to dynamic changes in multiple physiological systems, including hand movements along with heart signals. We anticipate seeing more research that uncovers such elegant real-time measurement of integrated nervous system activity (body and brain) that mobilizes during decision making.

A related area to be considered in future neuroscience work is to incorporate movement-based coding systems such as MPA, along with more traditional measures of decision-making style, which are typically based on self-report.

Indeed, there is little research on the relative distinctions between how people perceive themselves as decision makers, and their signature cognitive motivations detected by MPA that may elude self-reflection and self-perception. As consideration of both of these elements would feed into achieving a more complete understanding of the decision maker, we suggest that eventual convergence with neuroscience methods can begin to tease apart and map out the different components of decision-making style, and of the individual decision maker. It is likely that there are a number of neural networks that differentiate how we see ourselves as decision makers, and the motivations that drive our priorities as a decision maker.

## Conclusion

Our overall conclusion is that we are witnessing a new era of understanding not just about decision making, but about decision makers—and our traction is greatly aided by appreciating and assessing that people are embodied creatures who rely on movement to drive and navigate their own priorities as decision makers. We qualitatively self-navigate the quantitative mechanical world of distance, force, time and flow via movement, which serves essential functions in integrating the interplay between our internal states and our environment, and our sensorimotor integration that drives both thought and action. Future research that can sharpen our ability to acquire deeper insight into embodied decision making and decision makers would offer much in the way of optimizing human performance across many domains as well as guide therapeutic efforts that operate through a deep understanding of movement (Connors et al., 2018a).

## Author contributions

BC and RR equally contributed to conceptualizing the paper, reviewing references and materials, and writing and editing the submitted manuscript.

### Conflict of interest statement

The authors declare that the research was conducted in the absence of any commercial or financial relationships that could be construed as a potential conflict of interest.

## Footnotes

^1^“Cognition is embodied when it is deeply dependent upon features of the physical body of an agent, that is, when aspects of the agent's body beyond the brain play a significant causal or physically constitutive role in cognitive processing”: *The Stanford Encyclopedia of Philosophy*, plato.stanford.edu/entries/embodied-cognition.

^2^MPA can also be applied to videotaped recordings of an individual as captured in naturalistic settings, if the analyst can decipher a sufficient sampling of PGMs to support the crafting of the signature patterning of that individual.
